# Red Junglefowl Chicks Seek Contact With Humans During Foraging Task

**DOI:** 10.3389/fpsyg.2021.675526

**Published:** 2021-06-23

**Authors:** Diana Rubene, Hanne Løvlie

**Affiliations:** ^1^Department of Crop Production Ecology, Swedish University of Agricultural Sciences, Uppsala, Sweden; ^2^Department of Physics, Chemistry and Biology, Biology Division, Linköping University, Linköping, Sweden

**Keywords:** chicken, human-animal interactions, contact seeking, bird-human interactions, social cognition

## Abstract

Contact seeking with humans is documented in some domestic animals, mainly dogs, which have advanced communication skills. Domestication as a companion animal is thought to underlie this ability. However, also domesticated horses and goats display similar human-directed behaviors. This suggests either a broader effect of domestication on contact-seeking behavior, or alternatively, that social interactions with humans can result in the development of human contact seeking. As part of another study, we observed contact-seeking behavior in juvenile red junglefowl (*Gallus gallus*) chicks exposed to behavioral training since hatching, during a foraging task, where chicks were singly required to collect food rewards in a familiar arena using odor cues. If chicks left the arena, we recorded if they approached and looked up at the experimenter, or if they approached other objects (including another human). Chicks approached the experimenter significantly more often than they approached other objects. This behavior was not linked to a fast performance in the test arena, which gave some birds more time to explore the surroundings, or to learning ability measured in a cognitive task. Yet, the preference for the experimenter was lower for chicks that were handled more prior to the experiment. Also, approach probability was positively correlated with escape attempts in a novel arena test. The observed variation in approach behavior suggests a link to aspects of personality, and exposure to human interactions and experimental procedures. Our observations suggest that, although neither domesticated nor selectively bred, red junglefowl that are socialized with humans can potentially develop behavior used to describe contact seeking. Together with evidence from cognitive and behavioral studies, our results suggest that social experiences, not only domestication, can affect human-animal interactions. We propose how interactions between behavior, cognition and handling could be studied further in controlled settings to validate the preliminary findings of our study and uncover the underlying mechanisms.

## Introduction

The ability of animals to communicate with us influences our perception of their cognitive abilities and our attitudes toward our use of them in society (Nakajima et al., 2002; Knight and Barnett, 2008). Particularly, the social and emotional bonds humans share with their pet animals are reinforced by communication (Nagasawa et al., 2015), with dogs (*Canis familiaris*) and cats (*Felis catus*) being able to understand and respond to human signals (e.g., Miklósi et al., 2005). In general, behaviors such as approaching, and looking at another individual (also humans) are considered to be carried out with the intention of making contact with that individual. Approach to, and looking at, have therefore been used to measure contact-seeking in a range of species (e.g., Miklósi et al., 2003, 2005; Malavasi and Huber, 2016; Nawroth et al., 2016; Mastellone et al., 2020), and contact-seeking behavior has an important role for understanding heterospecific communication.

The propensity to display contact-seeking behaviors can differ between even closely related animal taxa. For example, when confronted with an unsolvable task, dogs appear to seek contact with humans by looking back at them, while wolves (*C. lupus*) do not (Miklósi et al., 2003). This seems to not be solely due to whether an animal is familiar with human interaction or not, as even wolves socialized with humans (Miklósi et al., 2003) and domestic cats (Miklósi et al., 2005) do not look at humans in such situations. On the other hand, domestic horses (*Equus caballus*) appear to seek human attention through eye contact and body language when they need help (Malavasi and Huber, 2016). Thus, both domestication, and selection as companion animals, could have shaped the development of these contact-seeking behaviors. Recently, however, domestic goats (*Capra aegagrus hircus*) were also shown to display similar contact-seeking behaviors (Nawroth et al., 2016). This suggests either a broader effect of domestication on human directed contact seeking than previously assumed, or alternatively, that, in some species, social interactions with humans can result in the development of human contact seeking (Gasci et al., 2009). That puppies display a lower degree of contact-seeking behavior compared to adult dogs indicates that these behaviors develop with age through increased social interactions with humans [but see Gasci et al. (2009) and Passalacqua et al. (2011)]. There might, thus, be multiple underlying processes behind the development of social human-directed behavior (Miklósi, 2009). Further studies are required to assess the relative contribution of domestication and selection, vs. social factors, such as the accumulation of social interactions with humans over the life of an individual, toward how likely individuals are to display such behavior.

So far, human-directed contact seeking has only been studied in mammals and our understanding of social interactions between humans and non-mammalian taxa, is poor. Birds, for example, are widespread and popular as pets, production animals and research subjects. Nevertheless, despite well-documented cognitive abilities of many bird species (e.g., ten Cate and Healy, 2017), human-directed contact-seeking behavior has, to our knowledge, not previously been documented. However, preference or filial behavior toward humans have in precocial birds been studied and interpreted within the concept of imprinting (Lorenz, 1937). Nevertheless, there is still a lack of investigation on human-directed behavior among birds.

In this study, we report human-directed approach behavior in a non-domesticated avian species, the red junglefowl (*Gallus gallus*), the ancestor of the domestic chicken (*G. gallus domesticus*). We measured how often birds, that had been exposed to positive human interactions during their development, approached and looked at human experimenters during a foraging task. This is a similar behavioral response as reported in other species as human contact seeking (Malavasi and Huber, 2016; Nawroth et al., 2016; Langbein et al., 2018; Mastellone et al., 2020). Then, we related variation in this behavior to individual birds‘ learning ability, amount of human handling received and their behavior in a novel arena test. Based on our results, we propose how interactions between behavioral traits related to personality and exposure to humans could be studied in a standardized setup to disentangle the underlying mechanisms of contact-seeking behavior.

Red junglefowl are well-suited for this study, because they are social, extensively used in behavioral research, and their cognitive abilities are well-developed and documented (Garnham and Løvlie, 2018; Marino, 2018). While not domesticated or selected for specific traits, they are still behaviorally and cognitively very similar to the domestic chicken (Garnham and Løvlie, 2018). Among lay people, fowl are considered to possess poorer cognitive abilities compared to mammals and even other bird species (e.g., corvids, Nakajima et al., 2002; Phillips and McCulloch, 2005). This has been refuted by recent studies [Marino, 2018; reviewed in Garnham and Løvlie (2018)], but likely still biases human understanding of the abilities and welfare requirements of these birds.

## Methods

### Study Population

The study was carried out during May-June 2019. The red junglefowl used belonged to a captive population kept at Linköping university. The population originates from birds wild-caught in Thailand (kept in Fröso Zoo, Sweden), and has been kept as research animals since around 2000 (for ca 15 generations), and pedigree-breed since 2011 (Sorato et al., 2018). Birds have not been bred for any specific behavioral or production-related traits. Until pedigree-breeding in 2011, the population was random bred. After 2011, breeding has aimed to increase genetic diversity in the population.

Sixteen red junglefowl chicks (7 weeks old, *n*_males_ = 9, *n*_females_ = 7) were used in the study. To enable individual recognition, chicks were wing-tagged with jiffy wing-bands (National Band and Tag Company). During their first 6 weeks after hatching, chicks were housed in 2 mixed-sex groups in cages (72 × 71 × 53 cm, L × W × H) with perches, heaters and light (12 h:12 h, 7–19 local time), and provided with food and water *ad libitum*, and checked on twice a day (morning and evening) by one of six researchers. Cages were cleaned, and new sawdust provided on a weekly basis. Prior to the present study, the chicks had been involved in two experiments. During day 2–14, innate olfactory preferences of chicks were tested in an unrewarded procedure. Thereafter, chicks performed associative learning and received food rewards (mealworms) during training, and to positively familiarize them with humans. Chicks were regularly picked up, held and spoken to by humans (e.g., during cleaning, when moved to test areas). Each chick spent ~20 min per day on 3 days per week in behavioral training. Behavioral training was done by a single experimenter (DR), although all chicks interacted with also other researchers, particularly during their first 14 days of life (details in Supplementary Table 1). All chicks were raised under the same conditions, and were exposed to the same set up and persons.

At the age of 6 weeks, chicks were moved to another facility where the present study took place. Chicks were housed in three mixed-sex groups in cages (124 × 76 × 84 cm, L × W × H) with perches and light (12 h:12 h, 7–19 local time, plus natural light from a window), and commercial poultry feed and water available *ad libitum*.

The study was carried out in accordance with Swedish ethical requirements (Linköping Ethical Committee, ethical permit numbers 288-2019).

### Personality Assay

At 4 weeks of age, we assessed the behavior of the chicks by testing them singly in a novel arena test (e.g., Favati et al., 2016; Zidar et al., 2018). The novel arena (114 × 76 × 40 cm; L × W × H) was divided into six equal, imaginary squares. To encourage exploration, an empty water bell was placed in the middle of every other square, obstructing the view of the full arena. The arena floor was covered in sawdust and a mesh roof was placed on top to prevent escapes. Boldness was measured as latency (in seconds) to move after being placed in the arena. If a chick did not move within 5 min, it received 5 min as a maximum latency for boldness and a mealworm was dropped ~15 cm in front of it to encourage movement. Once a chick started moving, activity and exploration were recorded for 7 min as the total number of square changes, and the latency to explore all six squares of the arena, respectively. Finally, we recorded the number of attempts made to escape the arena. Boldness, exploration, activity and escape attempts have been demonstrated to show repeatable variation in this population, thus are behavior that describe variation in personality (e.g., Favati et al., 2016; Zidar et al., 2017, 2018).

### Experimental Setup

Data for this study was collected during a foraging experiment, unrelated to the questions of the present study, and designed to investigate the responses of fowl to olfactory cues, described below. During the habituation stage of the experiment, it became evident that chicks appeared to display behavior used to measure contact-seeking in other species, toward the experimenter. Thus, we developed a protocol for recording this behavior throughout the experiment.

The experiment took part in a wall-less arena (180 × 180 × 0.1 cm, L × W × H), divided into nine squares by tape markings on the floor, within a test room (285 × 300 cm, with gray walls and floor, one door and a single window 1.5 m above floor level, Supplementary Figure 1). Thus, chicks were able to see, and move freely, within the test room and could interrupt their foraging behavior and leave the arena. Nine bowls (ø 5 cm) were placed in the arena, one in the center of each arena square (Supplementary Figure 1), and a food reward (mealworm) placed in each bowl. Birds were habituated to the arena, first in groups and then singly, until they foraged in a focused manner and showed no signs of anxiety.

### Foraging Experiment

The aim of the foraging experiment was to test if fowl show preference for food placed together with olfactory cues that they have previously learned (Rubene et al., in prep.). After habituation, each chick was individually presented with a task where it needed to use an olfactory cue to find food rewards. During the task, three of the bowls in the arena contained a food reward (mealworm) together with olfactory cue, three contained only food reward and three were empty. As fowl are primarily visual animals, using an olfactory cue to find food could be considered a challenging task, and some odors can be perceived as aversive [reviewed in Jones and Roper (1997) and Zidar and Løvlie (2012)]. During training and testing sessions, chicks showed no indications of stress (i.e., produced no distress vocalizations and freely approached the test set up). Each chick performed two test sessions; in the first session (“familiar odor session”), the odor was familiar (i.e., already associated with a food reward in earlier experiments) and, in the second session (“novel odor session”), the odor was unfamiliar. In the first session, an observer (KL) was present in the room as well as the experimenter (DR). This observer had provided the chicks with food and water several times weekly during their first 6 weeks of life, but had not been directly involved in behavioral training of them (Supplementary Table 1).

### Data Collection

During each test session, the behavior of each bird was observed for 5 min after it was introduced to the arena by the experimenter. For the purpose of the foraging experiment, we recorded the latency (in seconds) to first visit and the total number of visits to each arena square. For the present study, we recorded, through direct observation: (1) the number of times each chick exited the arena (i.e., moved outside the foraging arena by at least one body length), during each test session, and (2) whether during these exits the chick approached the experimenter within a distance of 0.5 m and looked up at the experimenter, whether they approached the observer, or if they approached other parts of the test room. We defined looking at experimenter as when the chick lifted and tilted its head such that the direction of the gaze was angled toward the eyes of the experimenter. This behavior was simple enough to be recorded directly, and as we were interested in observing behavior that may be indicative of contact seeking, it provided a more conservative measure than if we only recorded approaches. As we could not use camera recordings to precisely record gaze direction and duration of eye-contact, we did not score or analyze looking at human as a separate response, but only in combination with approaches. However, merely approaches have also been used to measure contact seeking (e.g., Mastellone et al., 2020).

The position of the experimenter in the room, relative to the arena, was alternated between individual birds and between sessions in a semi-random fashion (to obtain a balanced distribution), to avoid confounding effects of potential spatial preferences of the chicks (positions A, B, or C in Supplementary Figure 1). The observer was positioned at an adjacent position to the experimenter, in other words, if the experimenter was at A, the observer was at B, etc. The experimenter and observer always faced the chick during the test, but alternated their gaze between observing the chick and taking notes. When a chick exited the arena, it was encouraged to return to the task by the experimenter, both verbally and by body language. If a chick did not return to the arena within 30 s, it was picked up by the experimenter and placed at the starting position (marked in Supplementary Figure 1). When chicks exited the arena other than to approach the experimenter, they approached either the observer, a metal floor drain on the floor, or no specific object (Supplementary Figure 2).

Further data, observed in other studies on our test chicks, up to 9 weeks of age, was used, in order to explain variation in approach behavior: (1) time to retrieve all food from the foraging arena, as a measure of performance speed, (2) amount of handling (total number of training and testing trials each chick had experienced prior to the present study, where each trial involves being placed by a human in an arena and picked up again after making a choice), and (3) learning speed (in an olfactory discrimination task, measured as number of trials to pass, *sensu* e.g., Sorato et al., 2018), as a proxy for cognitive performance. Learning speed was available for 15 out of 16 chicks, which had successfully passed at least one associative learning task.

### Statistical Analyses

All analyses were carried out in R (R Core Team, 2019). The data were explored to confirm it was meeting assumptions of parametric statistics [e.g., collinearity, homogeneity of variance, according to protocol by Zuur et al. (2010)]. The behavioral measures activity and exploration were correlated (Spearman rank correlation, *r* = −0.65), thus, to avoid collinearity, we excluded exploration from further analysis. The number of times chicks exited the test arena in other direction than to the experimenter (including toward the observer) was very low; therefore, to avoid zero-inflated variable categories, we combined all other exits (i.e., not to experimenter) into a single category (“other direction”) prior to statistical analyses.

To investigate if chicks preferred to approach the experimenter or other objects (“other direction”) when exiting the arena, we used generalized linear models (GLM in package “lme4,” Bates et al., 2015) with a Poisson error distribution. As fixed factors we included sex (male or female), direction (“toward experimenter,” or “other direction”), session (“familiar odor session,” or “novel odor session”), experimenter position (A, B, or C, Supplementary Figure 1) and interactions between approach direction and sex, and approach direction and session. Given that previous studies reported the presence of sex differences in behavior of red junglefowl (e.g., Zidar et al., 2018), we included sex as a factor. We initially included chick ID as a random factor to control for multiple observations for each of the 16 birds (for each of the two sessions, we scored the number of approaches to experimenter and other exits). However, as this random factor explained zero variation, it was removed from the model. To simplify the model, non-significant effects and interactions were also removed if the removal resulted in a lower AICc value for the model. Model fit was assessed by comparing the AICc value of the full model to a null model (intercept only), with a cut-off at ΔAICc = 2.

To assess if seeking contact was linked to absence of food in the arena (i.e., chicks which retrieved all food faster might be more prone to leaving the arena), we used a GLM with Poisson error distribution. Number of approaches to the experimenter was used as response variable, and latency (in seconds) to retrieve all food rewards by the chick, was used as a fixed factor. Chick ID was included as random factor, and this time it was retained in the model. Model fit was assessed by comparing AICc value of the full model to a null model using the same cut-off as above.

To analyse potential links between behavioral responses describing variation in personality and contact-seeking behavior, we used a GLM with Poisson error distribution. We used the “number of times chicks approached the experimenter” as response variable, “session” and personality measures as fixed factors (“boldness,” “activity,” and “escape attempts”), and chick ID as random factor (which was again removed from model because it explained no variation). Again, non-significant effects were subsequently removed from the model, and model fit assessed by comparing AICc value of the full model to a null model as above.

To assess whether the preference for the experimenter (proportion of approaches) was related to the total amount of handling by humans each chick received, and/or learning speed, we used a quasibinomial GLM, to control for overdispersion in the data. “Proportion of approaches to experimenter” (out of all exits) were used as the response variable and “handling” and “learning speed” (averaged over performance in one task performed shortly before, and one shortly after the present study) as explanatory fixed factors. AIC cannot be calculated for quasibinomial models, therefore, we compared full model to a null model using Anova with a Chi-square test.

## Results

**Figure 1:**
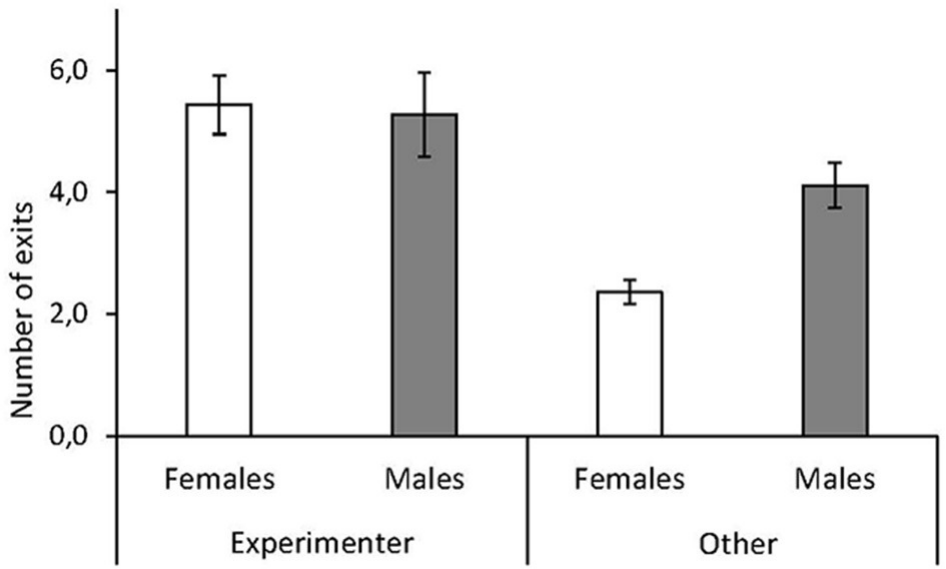
Sex differences in human contact-seeking by red junglefowl chicks. Mean number of exits per test session by female (white) and male (gray) chicks toward experimenter or in other direction, during a foraging task. Mean and standard error are given.

Red junglefowl chicks approached the experimenter when they exited the arena significantly more often than they exited in any other direction (approach experimenter: mean ± SD: 5.3 ± 2.3 times per session; other direction: mean ± SD: 3.3 ± 2.2 times per session, Table 1, Figure 1). We did not observe that chicks looked up at the observer, even when they approached this person. There were no overall sex differences, but a significant interaction between sex and approach direction revealed that males were more likely to approach other locations besides that of the experimenter, after leaving the arena, than females (mean ± SD, males: 4.1 ± 2.5; females: 2.3 ± 1.4 times per session, Table 1, Figure 1).

**Table 1:** Human contact-seeking by red junglefowl, during a foraging task; GLM results.

**Response**	**Fixed effects**	**Estimate**	**SE**	***Z***	***P***
All exits	Intercept	1.8	0.15	11.9	**<0.0001**
	Sex (male)	−0.05	0.15	−0.35	0.72
	Direction (other)	−0.83	0.21	−4	**<0.0001**
	Odor (novel)	−0.44	0.12	−3.6	**0.0003**
	Position (B)	0.07	0.15	0.49	0.62
	Position (C)	0.26	0.15	1.77	0.08
	Sex ^*^ Direction (male:other)	0.58	0.26	2.23	**0.02**
Approaches to experimenter	Intercept	1.60	0.15	11.00	**<0.0001**
	Escape attempts	0.14	0.06	2.13	**0.03**
	Odor (novel)	−0.29	0.15	−1.9	0.057

*The first model (“All exits”) tested differences in approaches to experimenter vs. other exits, in relation to sex, odor used in the test, and position of experimenter. The second model (“Approaches to experimenter”) assessed the influence of personality on contact-seeking behavior. Estimates, standard errors, z and p-values of fixed effects are presented from generalized linear models with Poisson distribution. Reference categories: sex = female, direction = experimenter, odor = familiar, position = A. Significant effects are indicated in bold. For all exits, ΔAICc = 25.60 between final model and null model; for approaches to experimenter, ΔAICc = 3.74 between final model and null model*.

**Figure 2:**
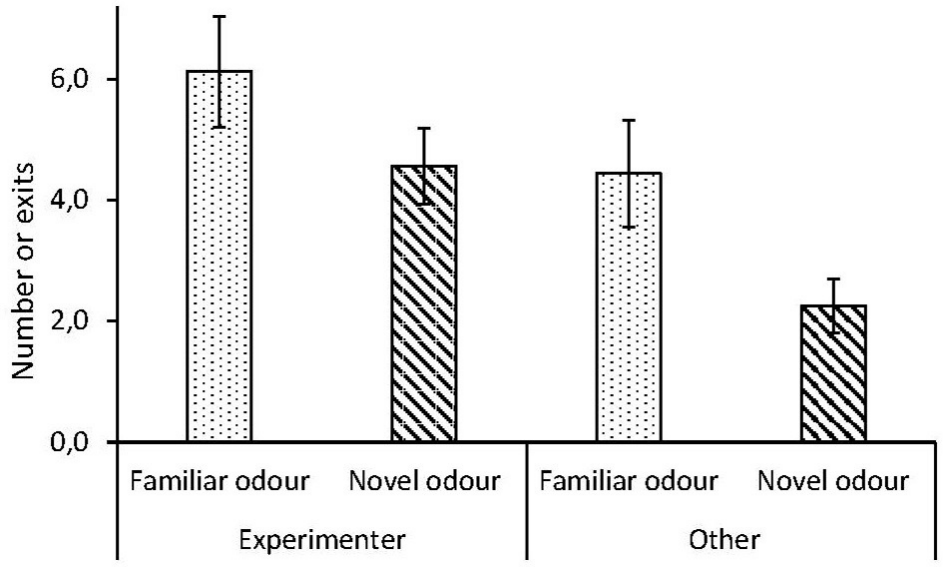
Human contact-seeking dependent on odor task, in red junglefowl chicks. Number of approaches per session by chicks toward experimenter or in other direction, in presence of familiar (dotted) or novel (striped) odor. Mean and standard error are given.

In the presence of a novel odor, chicks tended to exit the arena fewer times (mean ± SD: 3.4 ± 2.0 times per session), compared to when exposed to a familiar odor (mean ± SD: 5.3 ± 2.5 times per session). However, there was no interaction between session and the number of times chicks exited the arena, and chicks showed the same preference for the experimenter in the presence of a novel and a familiar odor (Table 1, Figure 2).

Chicks faster at finishing the foraging task did not approach the experimenter more often, as we found no effect of latency to retrieve all food from the arena on the number of approaches (estimate ± SE: −0.09 ± 0.08, *z* = −1.10, *p* = 0.26), and the full model did not differ significantly from the null model (ΔAICc = −0.96).

**Figure 3:**
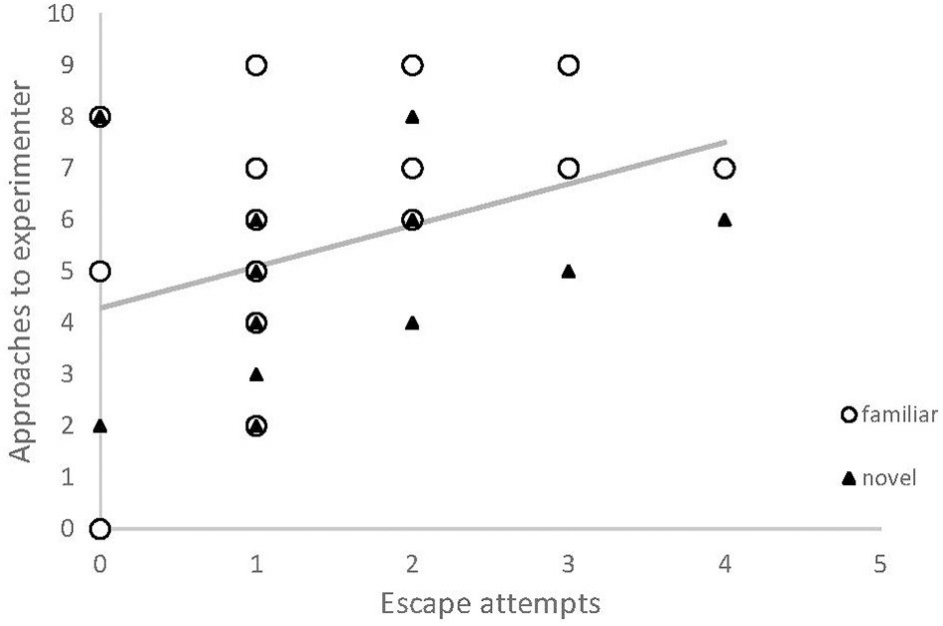
The relationship between human-contact seeking and behavioral measures used to describe personality, in red junglefowl chicks. Number of approaches toward experimenter was positively correlated with the number of escape attempts in a novel arena test. Data is from two test sessions (familiar odor: white circles; novel odor; black triangles). Regression line is based on the modeled relationship.

There was a positive correlation between number of times the experimenter was approached by a chick, and its number of escape attempts in a novel arena test (Table 1, Figure 3). No other behaviors measured in the novel arena assay explained variation in approach behavior.

**Figure 4:**
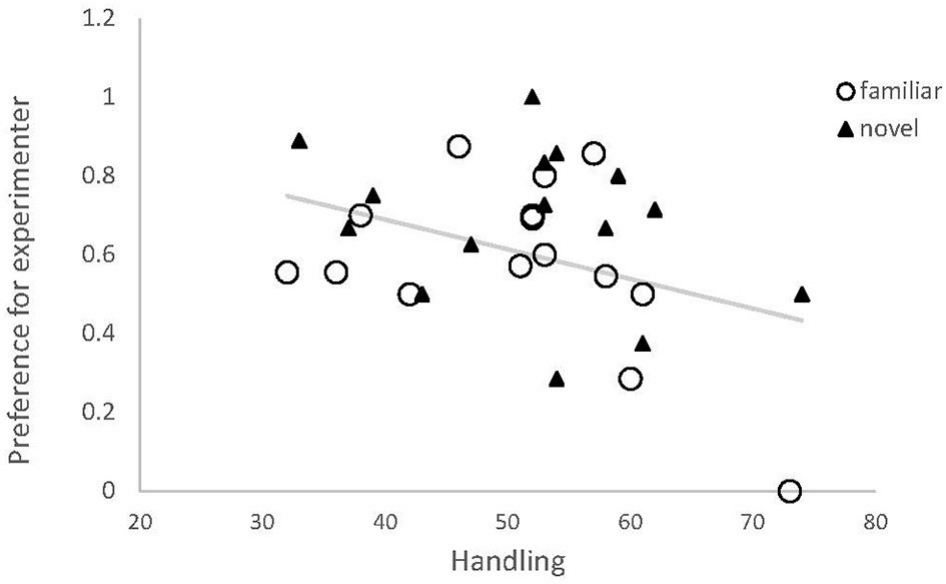
Preference for the experimenter by red junglefowl chicks, dependent on amount of handling by humans. Preference was expressed as proportion of approaches toward experimenter of all exits, and handling was the total number of training and test trials for each bird. Data from familiar odor session (black) and novel odor session (gray) are shown. Regression line is based on the modeled relationship.

During their first 7 weeks of life, chicks received between 32 and 74 handling/training trials (Supplementary Table 1). Learning speed among our test birds ranged from 14 to 44 trials, but did not explain the proportion of approaches made toward the experimenter (estimate ± SE: 0.01 ± 0.02, *t* = 0.72, *p* = 0.48), thus this factor was removed from the model. There was a negative correlation between preference for the experimenter and amount of handling received (estimate ± SE: −0.032 ± 0.015, *z* = −2.2, *p* = 0.036, Figure 4), and this final model differed significantly from the null model (df_Deviance_ = −6.32, *p* = 0.02).

## Discussion

In a task designed for another study, we observed contact-seeking behavior in red junglefowl chicks, measured as number of approaches toward the experimenter, combined with looking at the experimenter. This is similar to behaviors which have been considered to indicate contact seeking in other species (e.g., Malavasi and Huber, 2016; Nawroth et al., 2016; Mastellone et al., 2020). Analyses of this behavior showed that red junglefowl chicks, during a foraging task, were more likely to approach a human experimenter, with whom they had undergone behavioral training, than they were to approach other objects, including another human. In our chicks, contact-seeking behaviors occurred in similar frequencies in males and females, and in a familiar as well as in a novel odor environment. The propensity to approach the experimenter was unaffected by learning speed, negatively related to amount of human handling received and positively related to the number of escape attempts chicks made while in a novel arena. We discuss below the potential explanations of the observed patterns, and suggest how future studies should test the robustness of these preliminary findings.

We found that males were more likely to approach other objects than females (Figure 1), which may suggest that males were more exploratory. Yet, previous studies on this population of junglefowl have not found any sex differences in exploration behavior in novel environment contexts (e.g., Zidar et al., 2018). However, male zebra finches (*Taeniopygia guttata*) have been shown to be more prone to explore alone instead of with a conspecific partner compared to females (Schuett and Dall, 2009). Additionally, in a familiar arena setting, juvenile male junglefowl were more exploratory and spent more time pecking at food, while females spent more time in proximity of a conspecific stimulus bird (Väisänen and Jensen, 2003). Higher social reinstatement tendencies have also been found in female domestic chicks (Cailotto et al., 1989). Our results confirm that in familiar foraging settings male junglefowl appear to be more exploration-orientated, while females are more socially oriented, which probably explains the wider range of objects approached by male chicks and focus toward the experimenter by females.

Our chicks exited the arena and approached the experimenter fewer times in the presence of a novel odor, yet the level of preference for the experimenter did not differ significantly (Figure 2). A higher activity (number of exits) could be expected due to repeated testing in the same arena, as increased level of habituation can lead to increased exploration (Thys et al., 2017), and this could lead to a reduced need to seek contact. However, presence of a novel odor might induce novelty-aversion and fear responses (Jones and Roper, 1997), which could lead to higher contact seeking. Since we observed reduced activity, but no change in tendency to approach the experimenter, it suggests that the birds reacted to the change in odor, but it did not increase their fear level nor added to the perceived difficulty of the task.

### Influence of Handling, Cognitive Ability, and Personality

Unexpectedly, we found that the chicks' preference for the experimenter was negatively related to the amount of handling received by any human over their accumulated life up to testing. In imprinted domestic chicks, the exposure time has been found to be either unrelated (Suge and McCabe, 2004) or positively related (Bolhius, 1999) to preference. Also in goats, handling is positively related to human-directed behavior, but only if the animals are exposed to human interactions early in life (Langbein et al., 2018; Mastellone et al., 2020). In general, chicks which received most handling in our study were those that needed more time to successfully perform in various behavioral tasks. This might be due to poorer learning ability or due to behavioral issues, such as fear responses to humans or to isolation. We found no effect of learning ability on contact-seeking, and learning speed was not correlated to handling in our analysis, indicating that the observed pattern is not linked to variation in learning. Thus, it may appear that the most handled chicks were more fearful and therefore avoided approaching the experimenter. Yet, this is not what we experienced during training and handling of these chicks. In contrary, by the end of our learning experiment, the chicks that had been handled more appeared highly motivated and behaviorally comfortable with the experimental setup and human handling. Therefore, we instead propose that the most handled birds were more accustomed to both handling and isolation, thus they approached the test setup with less fear and more efficiency. Important in this context is that our experiment did not constitute an unsolvable task, thus, the food in arena was freely available to the birds, irrespective of whether they perceived olfactory cues as easy or difficult. Thus, the main difficulty of the task as perceived by the birds may not have been food retrieval, but something else, such as being placed alone in a large room (relative to home cages). In such case, it would be logical that the birds that have been more exposed to experimental procedures (in different test setups) experience the task as less challenging, leading them to seek contact less often.

Our findings also suggest a link between contact-seeking and aspects of behavior commonly used to describe personality. Individuals that attempted to escape the novel arena test more often also sought contact more often during the foraging task. Escape propensity could be due to higher fearfulness. However, in our previous work on the same population, escape propensity did not relate to other fear-related behavior, but instead seemed to describe a more proactive behavioral type (Zidar et al., 2017). On the other hand, those birds were not more exploratory, as would be expected of more proactive individuals. We believe that our chicks as a group were sufficiently socialized to display a significant preference for approaching the experimenter (Table 1, Figure 1), but variation in this preference within the group suggests that individuals that have received less training and exposure to experimental situations, and that are more fearful (or proactive) display more contact-seeking (Figures 3, 4). Previous studies on contact-seeking behaviors generally compare levels of behavior between treatment (socialized) and control groups, or between taxa (dog, wolf, and cat), yet tests on how the variation within the groups is influenced by different factors is scarce. Exploring interactions between different behavioral responses (e.g., fear and proactivity), handling and other potentially explanatory factors should be the focus of future research, which could further reveal whether individuals that are more likely to seek human contact do so because they are more fearful, or sensitive to isolation, have high social needs, or if they are more proactive.

### Eye Contact

Looking at the experimenter was included in our measure of contact-seeking behavior, similar to other studies (e.g., Miklósi et al., 2003, 2005; Malavasi and Huber, 2016; Nawroth et al., 2016; Mastellone et al., 2020). Direct eye contact is a powerful stimulus for most animals, including birds, which usually elicits flight and anti-predator responses (e.g., Rosa-Salva et al., 2007; Clucas et al., 2013). Wild birds react to human gaze with escape behavior (Clucas et al., 2013) and domestic chicks naïve to human gaze show fear responses (Rosa-Salva et al., 2007). As looking up at the experimenter in our chicks was combined with approach behavior, we find it unlikely that they perceived and monitored the experimenter as a potential threat. Some species, such as dogs and certain primates, may use eye contact to seek information from, or focus attention on, an experimenter for potential cues needed to obtain rewards (Thomsen, 1974; Savalli et al., 2016). We could speculate that the junglefowl in our study may have looked at the experimenter for similar reasons. Primates make less eye-contact with humans when placed in a novel cage, which could be explained by that more of their attentional space is taken up by the novelty (Thomsen, 1974). Our chicks made fewer exits from the arena in the presence of novel odor, yet we found no significant difference in their preference for the experimenter (Figure 2, Table 1), suggesting that a switch in attention toward novelty is a possible explanation for reduced activity. Using video recordings to obtain precise measurements of occurrence and frequency of eye contact and gaze direction could in future studies help to assess the direction of visual attention in birds under familiar/novel conditions.

### Underlying Mechanisms

Alternative explanations, besides contact seeking, could be proposed for why our chicks approached the experimenter during the foraging task. Newly hatched domesticated chicks show innate preferences to approach toward the head region of other animals and orientate themselves toward face-like configurations (Rosa-Salva et al., 2011, 2019). A possibility to why our chicks approached the experimenter more often could be because the experimenter constituted the most conspicuous object in the room. Preference for conspicuous objects is also known in newly hatched chicks (Bateson, 1966). Yet, these preferences for eyes, head-region, and conspicuousness are replaced with fear or avoidance responses later in development (Bateson, 1966). Considering the age of our chicks, an innate predisposition for conspicuous objects with a face as the main driver behind the observed behavior, seems unlikely. Additionally, in terms of conspicuousness, the observer should have been perceived as equally conspicuous, yet this person was largely ignored by our chicks (Supplementary Figure 2). The experimenter constituted a familiar object, and so did the other human (observer), although he was not familiar in a context of experimental procedures. Thus, the differences we observe in contact-seeking between the two familiar humans (both of whom were wearing same clothing during the experiment) may suggest that chicks could distinguish these two humans. Fowl can recognize individual conspecifics (e.g., Hauser and Huber-Eicher, 2004) but their ability to recognize individual humans has to our knowledge not been formally tested.

It is likely that our chicks associated the experimenter with an opportunity to obtain food rewards. Association with food underpins human-animal interactions, and food rewards are used during socialization training in most taxa (e.g., goats, Mastellone et al., 2020), and clearly also pet dogs and cats associate their owners with an opportunity to obtain food. The unsolvable task typically used to test contact seeking in different animal species is based on confronting an animal with inaccessible food, and contact-seeking behaviors directed toward humans are then interpreted as the animals seeking help to access the food (e.g., Miklósi et al., 2003, 2005; Malavasi and Huber, 2016; Nawroth et al., 2016; Mastellone et al., 2020). Thus, eliminating the association between humans and food might not be possible in this context. In an attempt to disentangle whether socialized wolves and dogs used food or play as motivation for interacting with humans, no evidence was found that the animals differentiated between these (Lazzaroni et al., 2020). Thus, the underlying motivation for contact seeking is still unclear, even in dogs or other domestic species (Lazzaroni et al., 2020; Mastellone et al., 2020). We assume that if our chicks only viewed the experimenter as a source of food, the individuals who were faster at retrieving all food rewards from the test arena, would approach the experimenter more often, as they had more time to become unmotivated. We did not find any evidence for this. In this and previous experiments, the birds did not receive food directly from the experimenter, but always had to retrieve it from the experimental setup, wherefore they had little reason to view the experimenter as a direct source of food. Thus, we suggest that the association of experimenter with food was similar to that displayed by other taxa, and we may consider that human-directed behaviors displayed by socialized animals in challenging situations might be similar among a wider range of species than previously documented.

Another mechanism, which may contribute to why there was a preference for our birds to show contact-seeking toward the experimenter, is filial imprinting (Lorenz, 1937; Bateson, 1966; Hess, 1973). Imprinting can result in behavior similar to contact seeking, such as the tendency to approach an object, which is typically displayed in precocial birds. The ability to imprint has been documented in birds up to 10 days of age (Gaioni et al., 1978), and our chicks were exposed to humans during this time period. However, it is well-documented that filial imprinting is weakened by exposure to conspecifics (Town, 2011) and by age (Gaioni et al., 1978), and is eventually lost if repeated exposure occurs in absence of positive reinforcement stimuli (Salzen and Sluckin, 1959). Our birds were reared in conspecific groups from hatching, exposed repeatedly, but only shortly, to several humans, and did not receive food rewards during their first 2 weeks of life (Supplementary Table 1). These aspects together would suggest that they were most likely imprinted on their conspecifics, and that imprinting on the experimenter is unlikely to have occurred during this early life period.

Recently shown, the presence of positive reinforcement (e.g., food, brooding) can through associative learning prolong the effect of imprinting on behavior (domestic chicks; Junco, 2017). Alternatively, early exposure to humans during a sensitive period (when also imprinting normally occurs), may have facilitated learning and development of preference for the experimenter later in development (Bateson, 1966; Yamaguchi et al., 2012). This may happen *via* memory-priming, a process mediated by release of thyroid hormone (T3) that controls the start and end of sensitive period in domestic chicks (Yamaguchi et al., 2012). Human-directed behaviors in goats, which are also precocial animals, can only be increased by socialization if the interactions start early in life (Mastellone et al., 2020). A similar mechanism may have created the preference for experimenter observed in our chicks, by early exposure, repeated interactions and reinforcement by food rewards. Red junglefowl become independent from their mother around the age of 10–12 weeks (Collias et al., 1994), and at the time of our study, they were 7 weeks old. As no study has documented occurrence of imprinting at such late age or its maintenance for such a long time, we find it less likely that the chicks were imprinted on the experimenter and more likely that early exposure to humans in general facilitated development of positive association with the experimenter through repeated social interactions. Other animal species display behaviors similar to imprinting during social bond formation early in life, and there are clear “similarities between imprinting and socialization in non-precocial animals (e.g., apes)” (Hoffman and Ratner, 1973). Early experience during sensitive periods essentially determines the animals behavior and ability to socialize with conspecifics and humans (Dietz et al., 2018). Additional controlled experiments comparing birds who are exposed to social interactions with birds who only receive standard exposure through daily feeding are needed to confirm this. In addition, testing how adult birds respond to socialization training depending on whether they have been exposed to humans early in life would increase our understanding of effects of imprinting, repeated interactions and age on human-directed behaviors. Future research should therefore further explore potential for contact seeking in birds, together with the details of its underlying mechanisms.

### Domestication

Our observations suggest that a species that is neither domesticated nor selected for human companionship has the potential to develop behaviors associated with seeking social contact with humans. In our birds, this was possibly a result of early exposure to humans, which facilitated development of positive association later in life through repeated social interactions with humans. In dogs, both genetic and social factors can influence human-directed contact seeking (Passalacqua et al., 2011; Udell et al., 2014; Persson et al., 2015). Domestication is generally acknowledged as the main driver of the development of animal-human communication skills, through multiple processes, such as selection, as well as frequent social interactions (Miklósi, 2009). Human-directed contact seeking has been reported in other domestic species (horses; Passalacqua et al., 2011; goats; Nawroth et al., 2016). The population of red junglefowl in the current study has been kept in captivity for over 15 generations, but is not to be considered domesticated. This is because no intentional nor directional selection have been carried out on any specific trait (pedigree breeding started 2011, only to reduce inbreeding). Human contact (beyond brief daily contact with game keepers) has depended on the experiments that have been carried out across the years, none that have focused on human contact seeking nor similar. Thus, we conclude that the contact-seeking behavior observed in our study is most likely not explained by selection history of the population.

Based on the current state of knowledge, it appears that early interactions with humans can lead to human-directed social behaviors in species that are domesticated and either precocial or group-living (dogs; Miklósi et al., 2003; goats; Nawroth et al., 2016; horses; Malavasi and Huber, 2016; Mastellone et al., 2020). Only domestication (cats; Miklósi et al., 2005) or only group-living (wolves; Miklósi et al., 2003) seems to be associated with clearly less developed human-directed behaviors, even when these animals are raised under same conditions as dogs. Junglefowl are not domesticated, but the fact that they are precocial and group living may make them predisposed to developing social interactions with humans. Experiments with a wider range of species are needed, to determine which factors contribute toward the observed variation between species in their propensity to display human-directed contact seeking.

### Implications

Birds, in general, and fowl, in particular, are popular subjects in behavioral research, much of which involves human observers. The possibility that also birds pay attention to human cues, suggests that human observers might unintentionally affect their behavior [through presence, position or even gaze direction, e.g., Rosa-Salva et al. (2007)]. These potential human observer effects on bird performance and behavior should be controlled for by accounting for factors like observer position and gaze direction during experiments, or analyzing behavior through video recordings rather than direct observation. How animals respond to observers may be biased by observer identity [e.g., farm animals perceive interactions with humans as negative, neutral or positive, and respond with different behavior depending on the person and their routine, reviewed in Hosey and Melfi (2014)], thus observer identity should be included in analysis when more than one observer is used. While social relationships between research animals and their experimenters may make interpretation and analysis of experimental data more complicated, these relationships can have positive implications. For example, humans interacting with primates and felids, by playing with and talking to them seems to promote behaviors indicative of increased welfare [reviewed in Hosey and Melfi (2014)].

Recent research into cognition, personality and behavior of fowl have considerably advanced our understanding of their cognitive skills and sophisticated behavior [reviewed in Nicol (2015), Marino (2018), and Garnham and Løvlie (2018)]. These findings call for rethinking the image of chickens as having poor cognitive abilities, which in turn have implications for our use of these animals and our understanding of their welfare needs. In the poultry industry, domestic fowl suffer poor conditions with limited opportunity to perform natural behaviors and social interactions (Prescott et al., 2003; Nicol, 2015). Our study improves the understanding of animal-human interactions and opens the possibility for that the social abilities in fowl may be more complex than previously perceived; for example, it demonstrates that they may be capable of heterospecific communication, which should further improve our appreciation of them.

## Data Availability Statement

The original contributions presented in the study are included in the article/Supplementary Material, further inquiries can be directed to the corresponding author/s.

## Ethics Statement

The animal study was reviewed and approved by Linköping Ethical Committee, ethical permit numbers 288-2019, Linköping, Sweden.

## Author Contributions

DR ad HL conceived the idea and wrote the manuscript. DR carried out behavioral observations and analyzed the data in discussion with HL. Both authors contributed to the article and approved the submitted version.

## Conflict of Interest

The authors declare that the research was conducted in the absence of any commercial or financial relationships that could be construed as a potential conflict of interest.
