# Supplementary material for: Red Junglefowl Chicks Seek Contact With Humans During Foraging Task
Source: Front Psychol. 2021 Jun 23;12:675526. doi: 10.3389/fpsyg.2021.675526 (PMC8260840; doi:10.3389/fpsyg.2021.675526)
Supplement: Supplementary file 2 [file Data_Sheet_1.PDF]

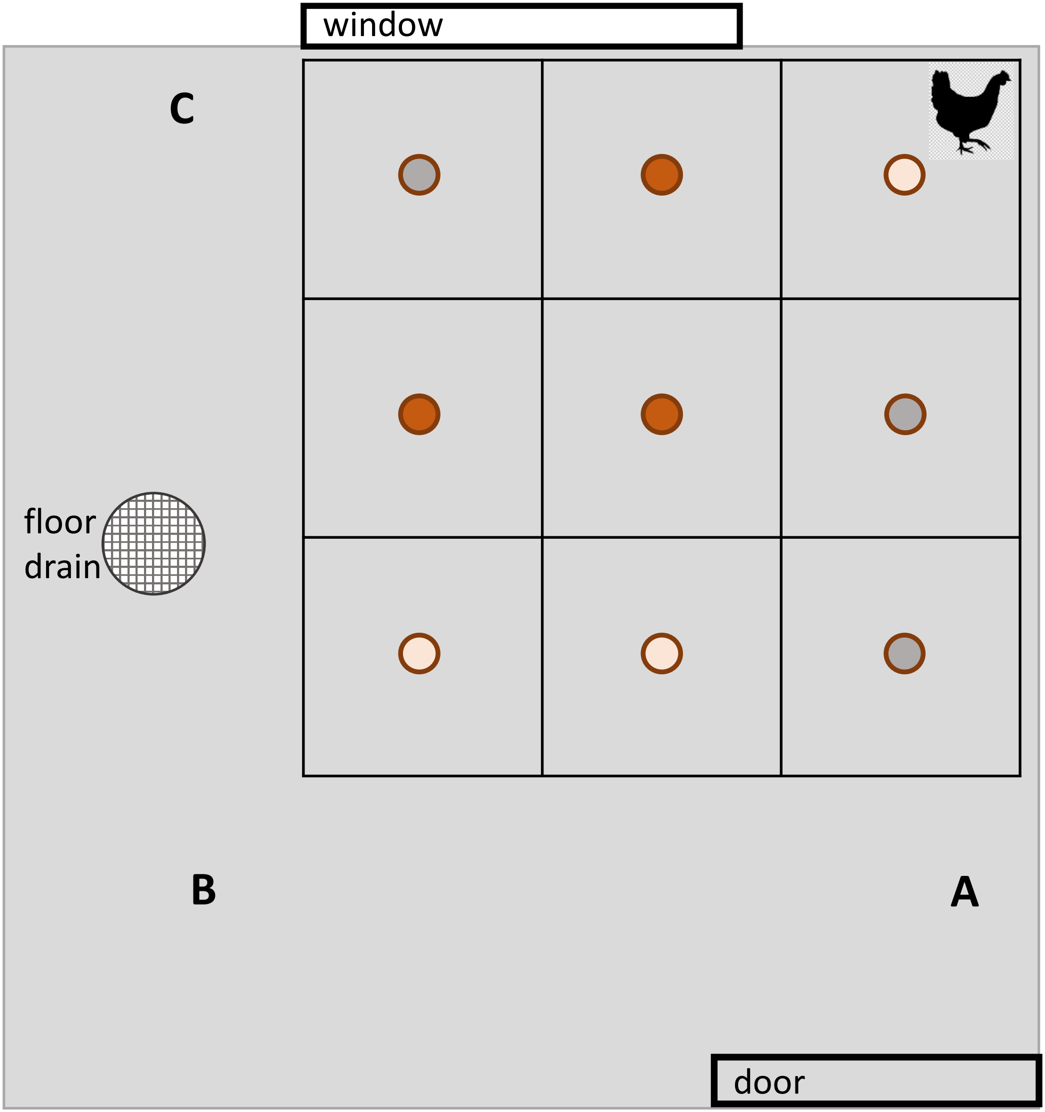

Figure S1. Test room with foraging arena, borders marked with tape on the floor. Chick release position illustrated with an image, circles show positions of food bowls containing different stimuli+rewards, and experimenter positions indicated with letters A-B-C.
