# Supplementary material for: Red Junglefowl Chicks Seek Contact With Humans During Foraging Task
Source: Front Psychol. 2021 Jun 23;12:675526. doi: 10.3389/fpsyg.2021.675526 (PMC8260840; doi:10.3389/fpsyg.2021.675526)
Supplement: Supplementary file 3 [file Data_Sheet_2.PDF]

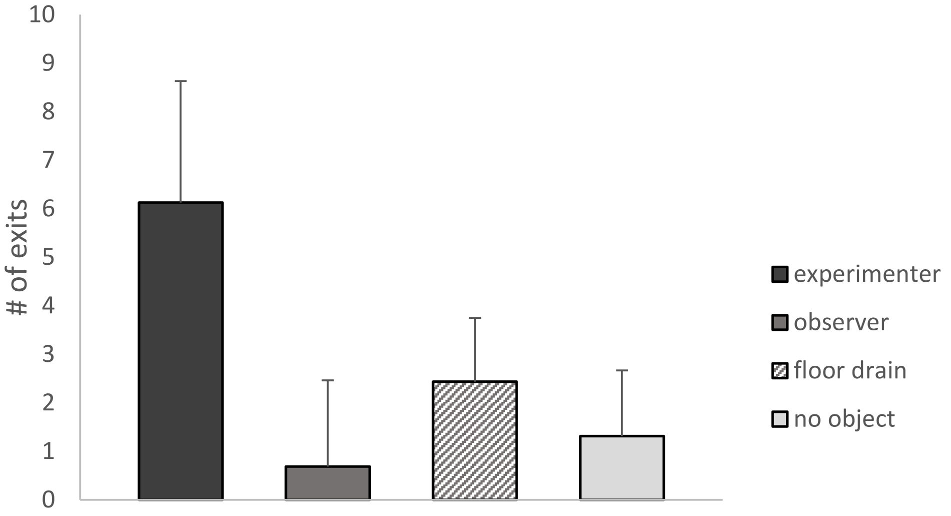

Figure S2. Number of exits from the test arena by chicks during the first test session (familiar odor session). Mean number of exits with standard deviation are shown, towards the experimenter, the observer, a floor drain and no specific object. The data for all categories other than the experimenter were pooled prior to analysis.
