# Supplementary material for: Red Junglefowl Chicks Seek Contact With Humans During Foraging Task
Source: Front Psychol. 2021 Jun 23;12:675526. doi: 10.3389/fpsyg.2021.675526 (PMC8260840; doi:10.3389/fpsyg.2021.675526)
Supplement: Supplementary file 4 [file Data_Sheet_3.PDF]

Table S1. All procedures that the red junglefowl chicks participated in, from hatching to the age of 7 weeks, identity of humans involved (initials), and exposure (number of trials) per bird. Initials of the experimenter (DR) and the observer (KL) who participated in the foraging experiment are marked in bold.

**Age (weeks)**	**Procedure**	**Reward**	**Human**	**Exposure per bird**
0	Hatching, tagging, sorting into cages	none	**KL**<br>LG<br>EA<br>SGD<br>HL	once
0-2	Feeding, cleaning	none	**KL**<br>**DR**<br>LG<br>EA<br>SGD	daily
0-2	Olfactory preference	none	**DR**<br>EA	6-18 trials
2-6	Associative learning	mealworm	**DR**	24-61 trials
4	Novel arena test	none	**KL**<br>EA	1 trial
6	Move to new facility	none	**DR**<br>EvP	once
7	Habituation to foraging arena	mealworm	**DR**	3-4 trials
7	Foraging experiment	mealworm	**DR**<br>**KL**	2 trials
